# Prevalence of *Yersinia enterocolitica* Bioserotype 3/O:3 among Children with Diarrhea, China, 2010–2015

**DOI:** 10.3201/eid2309.160827

**Published:** 2017-09

**Authors:** Ran Duan, Junrong Liang, Jing Zhang, Yuhuang Chen, Jing Wang, Jing Tong, Bangcheng Guo, Wanfu Hu, Mingliu Wang, Jiayong Zhao, Chang Liu, Huijing Hao, Xin Wang, Huaiqi Jing

**Affiliations:** State Key Laboratory of Infectious Disease Prevention and Control, National Institute for Communicable Disease Control and Prevention, Chinese Center for Disease Control and Prevention, Beijing, China (R. Duan, J. Liang, J. Zhang, Y. Chen, C. Liu, H. Hao, X. Wang, H. Jing);; Dongcheng Centre for Disease Control and Prevention, Beijing (J. Wang);; Xuzhou Municipal Centre for Disease Control and Prevention, Xuzhou, China (J. Tong);; Ningxia Hui Autonomous Region Center for Disease Control and Prevention, Yinchuan, China (B. Guo);; Anhui Provincial Center for Disease Control and Prevention, Hefei, China (W. Hu);; Guangxi Zhuang Autonomous Region Center for Disease Control and Prevention, Nanning, China (M. Wang);; Henan Provincial Centre for Disease Control and Prevention, Zhengzhou, China (J. Zhao)

**Keywords:** *Yersinia enterocolitica*, 3/O:3, China, infectious diarrhea, children, diarrhea, zoonoses, bacteria, bacillary dysentery, enteric infections, gastroenteritis, PFGE pattern, yersiniosis, fecal leukocytes, stool

## Abstract

*Yersinia enterocolitica* is thought to not significantly contribute to diarrheal disease in China, but evidence substantiating this claim is limited. We determined the prevalence of *Y. enterocolitica* infection and strain types present among children <5 years of age with diarrhea in China. The overall prevalence of pathogenic isolates was 0.59%. Prevalence of pathogenic bioserotype 3/O:3 varied geographically. In this population, the presence of fecal leukocytes was a characteristic of *Y. enterocolitica* infection and should be used as an indication for microbiological diagnostic testing, rather than for the diagnosis of bacillary dysentery. In contrast with *Y. enterocolitica* isolates from adults, which were primarily biotype 1A, isolates from children were primarily bioserotype 3/O:3. Most pathogenic isolates from children shared pulsed-field gel electrophoresis patterns with isolates from pigs and dogs, suggesting a possible link between isolates from animals and infections in children. Our findings underscore the need for improved diagnostics for this underestimated pathogen.

*Yersinia enterocolitica* is an emerging infectious pathogen that has caused wide public health concern since the 1980s. After campylobacteriosis and salmonellosis, yersiniosis ranks third most common among the notifiable bacterial zoonoses in the European Union (*1*,*2*). The incidence of human yersiniosis was 1.92 cases/100,000 population in 2013 in Europe (*3*); in the United States, incidence decreased from 1.0 cases/100,000 population in 1996 to 0.3 cases/100,000 population in 2009 (*4*). Gastroenteritis and enteritis are among the most common clinical signs. Autoimmune complications such as reactive arthritis sometimes occur (*2*,*5*). Deadly hemorrhagic septicemic yersiniosis occurs in immune-compromised patients. Strains of *Y. enterocolitica* biotype 1A (1 of the 6 biotypes) lack the pYV plasmid and the major chromosomal determinants of virulence and, thus, have been regarded as avirulent (*2*). However, this avirulent biotype has also been implicated in foodborne and nosocomial outbreaks and has reportedly produced disease symptoms indistinguishable from those produced by the known pathogenic biotypes (*6*–*8*).

In most countries in Europe, the bioserotype 4/O:3 accounts for ≈80% of human infections; 4/O:3 is also dominant in North America, where 3/O:3 infection is rarely reported (*9*). Conversely, 3/O:3 is the most prevalent bioserotype in China (*10*–*15*). Studies have shown that the prevalence of pathogenic strains among pigs in China is higher than that in countries of Europe (*15*,*16*). However, except for 2 outbreaks reported in the 1980s (*10*), we have little data concerning human infections in China. Because yersiniosis is not notifiable through the national surveillance systems in China, most hospitals do not routinely tested for *Y. enterocolitica*. In China, infectious diarrhea is primarily diagnosed on the basis of clinical examination rather than microbiological diagnostic testing (except for rotavirus, norovirus, and a few types of bacteria in some large hospitals). For example, shigellosis is often diagnosed in persons with signs such as tenesmus after leukocytes are found in their fecal samples. These diagnostic criteria render *Shigella*, *Salmonella*, enteroinvasive *Escherichia coli*, *Campylobacter*, and *Y. enterocolitca* infections indistinguishable.

According to surveys around the world, most *Yersinia* infections have occurred in infants and young children (*17*,*18*). In the United States, ≈100,000 episodes of foodborne illness caused by *Y. enterocolitica* occur annually, and risk for disease is higher among infants (*4*,*19*). In Germany, the average annual incidence of *Y. enterocolitica* infection among children <5 years of age was ≈12-fold higher than the average incidence among persons >5 years of age (*3*,*20*). Thus, in 10 regions of China, we performed microbiological diagnostic tests for children <5 years of age with diarrhea to determine the prevalence of *Y. enterocolitica* infection in this population and the need for improved diagnosis of yersiniosis. We also investigated possible links between strains from animals and humans.

## Methods

### Population Design and Collection of Case Information and Samples

During 2010–2015, we invited all patients with diarrhea from 17 hospitals to participate in this study. Diarrhea was defined according to the Global Enteric Multicenter Study: >3 loose stools within the previous 24 h (*21*). The study participants provided informed consent, fecal samples, and completed questionnaires. We followed the same protocol for all cases and excluded cases if either sample or questionnaire was lost.

### Sampling from Children

We recruited children <5 years of age with diarrhea at sentinel pediatric hospitals in different parts of China: Henan in central China; Beijing and Tianjin in northern China; Jiangsu, Shandong, and Anhui in eastern China; Guangxi in southern China; Sichuan and Yunnan in southwestern China; and Ningxia in northwestern China. Within each region, we gave primary hospitals (such as community hospitals in cities and village clinics in the countryside) the opportunity to become sentinel sites for this study. The staff of sentinel hospitals were capable of collecting case information and specimens and taking into account patients’ environment, folk customs, and eating habits during treatment. The same procedures were performed at each site to avoid bias in sampling procedures and in storing and handling samples. In some village clinics, fecal microscopy could not be conducted.

To compare the *Y. enterocolitica* prevalence between children and adults, we collected samples from 2 sites in central Beijing. We recruited adults from a general hospital and children from a pediatric hospital 5 km away that was also 1 of the sentinel hospitals for this study.

### Questionnaire

The questionnaire included questions about demographics (name, sex, birth date, address, and contact information) and clinical features (date of onset, date of visiting doctor, diarrhea frequency, body temperature, vomiting, fecal characteristics, and results of routine fecal sample inspection). Fecal samples were routinely examined for the presence of leukocytes and erythrocytes. Doctors wrote the primary diagnosis on the patient’s questionnaire.

### Sample Collection

Fresh fecal samples were collected from patients after enrollment in the study. Fecal samples were stored in peptone sorbitol bile broth (Fluka, Everett, WA, USA) at 4°C.

### *Y. enterocolitica* Isolation and Identification 

During the study, we conducted 2 technical trainings for sentinel hospital staff on *Y. enterocolitica* isolation and identification. *Y. enterocolitica* was isolated from samples by following the procedures described previously (*15*). To ensure laboratory capacity, we sent for assessment samples to the sentinel hospital staff who were blinded to sample identity. Hospital staff enriched the strains in peptone sorbitol bile broth at 4°C for 21 d and then amplified 2 *Y. enterocolitica* genes: *foxA* (conserved) and *ail* (pathogenic) (*22*). Samples positive for either or both of these genes were inoculated onto Yersinia Selective Agar (Schiemann’s CIN [Cefsulodin, Irgasan, Novobiocin] agar; Oxoid, Basingstoke, UK). To obtain pure cultures, staff then inoculated the presumptive *Y. enterocolitica* colonies having a typical bull’s-eye appearance on CIN agar onto brain–heart infusion agar plates and incubated them at 25°C for 24 h (*10*). Hospital staff performed the biochemical test Analytical Profile Index (API) 20E (bioMérieux, Marcy l’Etoile, France) and bioserotype identification methods reviewed by Wang et al. with all isolates (*13*). The Wauters’ biotype method was used (*23*).

### Identification of Pathogenic Strains and Cluster Analysis

We amplified virulence genes (*ail*, *ystA*, *ystB*, *virF*, and *yadA*) from the chromosomes and plasmids for all *Y. enterocolitica* isolates. We used the PCR method, including primer sequences and annealing temperatures, described by Liang et al. (*15*).

For the analysis of identified pathogenic isolates, we used the pulsed-field gel electrophoresis (PFGE) method described by Wang et al., with the following modifications: the DNA samples were digested with 25 U *Not*I and electrophoresed with pulse times from 2 to 20 s over 18 h at 200 V (*13*). For data analysis, we imported the images of gels into the PFGE database of *Y. enterocolitica* strains from China and performed a cluster analysis for the serotypes O:3 and O:9. The clustering of band patterns was performed by using BioNumerics software version 5.1 (http://www.applied-maths.com/bionumerics) and the Pearson algorithm. We visually inspected all patterns after computer analysis. For patterns that were indistinguishable by computer and visual inspection, we assigned a pattern designation.

## Results

### Characteristics of Pathogenic *Y. enterocolitica* Infection among Children

#### Prevalence and Demographics

**Figure 1:**
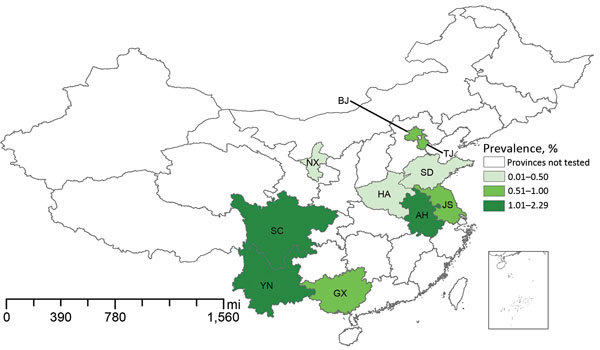
Prevalence of pathogenic *Yersinia enterocolitica* infection among children <5 years of age with diarrhea, by region, China, 2010–2015. Inset shows the islands of China in the South China Sea. AH, Anhui; BJ, Beijing; GX, Guangxi; HA, Henan; JS, Jiangsu; NX, Ningxia; SC, Sichuan; SD, Shandong; TJ, Tianjin; YN, Yunnan.

**Figure 2:**
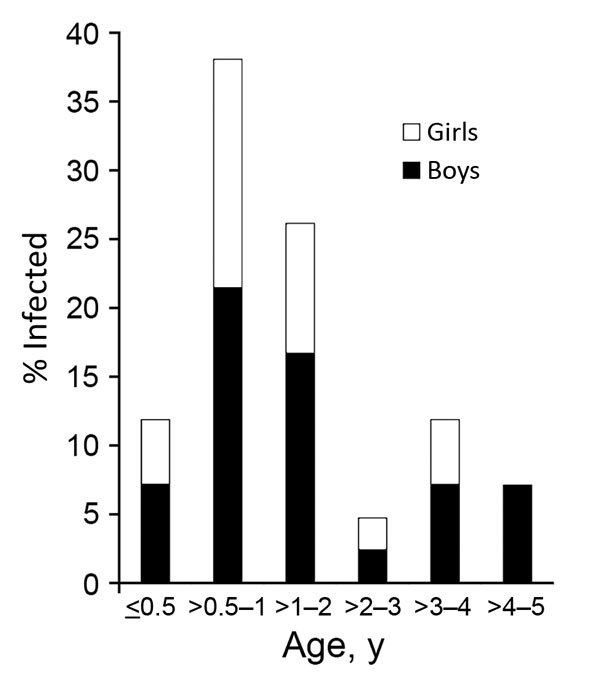
Percentage infected with pathogenic *Yersinia enterocolitica*, by age and sex, of total infected with *Y. enterocolitica*, China, 2010–2015.

We recruited a total of 7,304 patients <5 years of age with diarrhea from 10 regions of China. Fecal samples and answered questionnaires were collected for each patient, but 18 were excluded because either sample or questionnaire was lost. In total, we found 43 patients with pathogenic *Y. enterocolitica* infection. The average prevalence of *Y. enterocolitica* disease in all 10 regions was 0.59% (43/7,304); prevalence was highest in Anhui Province (2.29%, 3/131). *Y. enterocolitica* prevalence among young children with diarrhea was generally classified into 3 levels: 0.01%–0.50% (Shandong, Ningxia, and Henan); 0.51%–1.00% (Beijing, Guangxi, Tianjin, and Jiangsu); and 1.01%–2.29% (Anhui, Yunnan, and Sichuan) (Figure 1). Through year-round collection, we found that cases of pathogenic *Y. enterocolitica* infection occurred during January–November. The prevalence calculated for southern China (0.80%) was slightly higher than that for northern China (0.53%), when the northern and southern regions were defined by the Huaihe River, the natural border. Cases occurred more often in boys than in girls (1.63:1) (Figure 2). We found the largest proportion of *Y. enterocolitca* infections among children >0.5–2 years of age; among children in this age group, more cases also occurred in boys than in girls (1.45:1).

### Fecal Characteristics

**Figure 3:**
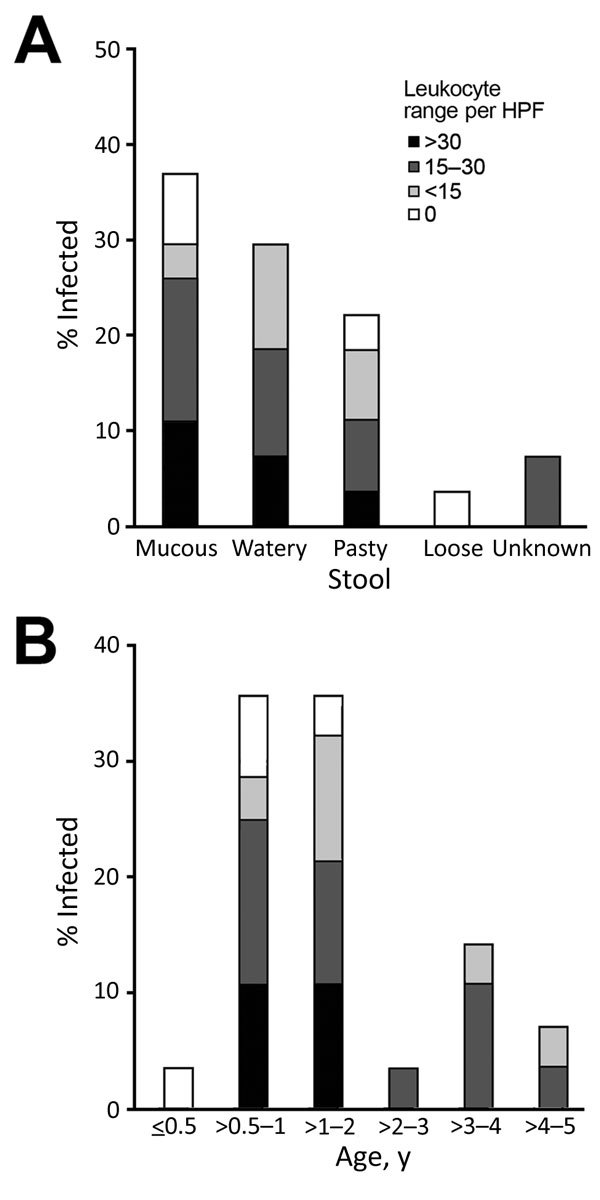
Fecal leukocyte ranges among children <5 years of age infected with pathogenic *Yersinia enterocolitica*, by fecal characteristics (A) and age (B), China, 2010–2015. HPF, high-power field.

Fecal samples from children <5 years of age infected with pathogenic *Y. enterocolitica* had the following characteristics: mucous (37%), watery (30%), pasty (22%), and loose (4%) (Figure 3, panel A). Fecal microscopy was performed with fecal samples from all children; leukocytes were detected in samples from 85% (23/27) of children <5 years of age with diarrhea. A higher proportion of the fecal samples from those in the >0.5–2 years age group had leukocyte counts >15 cells/high-power field (HPF). Fecal leukocyte counts were >30 cells/HPF only among patients in this age group, and in 2 cases the concentration reached as high as 45 cells/HPF and 84 cells/HPF.

### Bioserotypes of Isolates from Patients with Acute Diarrhea and Prolonged Shedding 

The predominant cause of acute *Yersinia* infection among children <5 years of age was bioserotype 3/O:3 (Table); 41 of 43 patients were infected with this bioserotype. The other 2 patients were infected with 4/O:3 or 2/O:9, both found in Beijing. Except for one 3/O:3 infection, all isolates harbored the *Yersinia* virulence plasmid and virulence genes *ail*, *ystA*, *virF*, and *yadA*. In addition to the acute diarrhea cases, 3 cases from different regions involved prolonged *Y. enterocolitica* 3/O:3 shedding that had progressed from acute diarrhea. These patients were 1–1.5 years of age. Once pathogen shedding stopped, the diarrhea ceased as well. The period of shedding could be as long as ≈3 months.

**Table:** Bioserotype and virulence genes of pathogenic *Yersinia enterocolitica* isolates from children <5 years of age with diarrhea, China, 2010–2015

Bioserotype	No. cases	*ail*	*ystA*	*ystB*	*yadA*	*virF*
3/O:3	40	+	+	–	+	+
	1	+	+	–	–	–
4/O:3	1	+	+	–	+	+
2/O:9	1	+	+	–	+	+

### Difference in *Y. enterocolitica* Prevalence between Children and Adults

A total of 2,127 children and 1,904 adults with diarrhea were enrolled at the Beijing sites. Pathogenic *Y. enterocolitica* infection accounted for 0.61% (13/2,127) of the children and 0.11% (2/1,904) of the adults tested. One child and 1 adult had 2/O:9 *Y. enterocolitica* infections; the other 13 patients had 3/O:3 infections. Leukocytes were detected in the fecal samples of all 13 children and 1 of the 2 adults.

**Figure 4:**
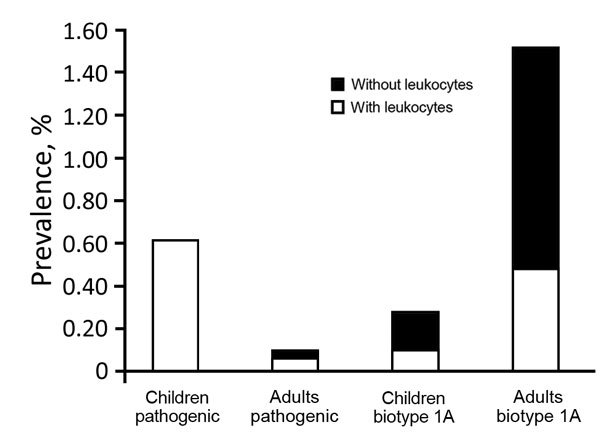
Prevalence of pathogenic and biotype 1A *Yersinia enterocolitica* infection among children <5 years of age and adults with diarrhea, by leukocyte positivity, Beijing, China, 2010–2015.

The overall prevalence of *Y. enterocolitica* biotype 1A was 0.28% among children (6/2,127) and 1.52% among adults (29/1,904) (Figure 4). Among the 35 patients with biotype 1A infections, we found leukocytes in the fecal samples of 33% (2/6) of children and 31% (9/29) of adults. Regardless of whether the samples had leukocytes or not, all isolates (6/6) from children and most isolates (20/29) from adults carried the *ystB* gene.

### PFGE Analysis of Pathogenic *Y. enterocolitica* Isolates from Children and Animals

PFGE patterns for most of the pathogenic isolates from children (36/43, 84%), including the one 2/O:9 isolate, were indistinguishable from those of isolates from pigs and dogs (data not shown). The rest of the isolates (7/43), including the one 4/O:3 isolate, did not share a pattern with any bacteria isolates from these animals. Isolates from children, pigs, and dogs displayed many patterns, and some patterns appeared in bacteria isolated from multiple hosts in >1 region. We found the predominant patterns K6GN11C30021 and K6GN11C30012 of the 3/O:3 bioserotype (shared by isolates from children, pigs, and dogs) in 67% (24/36) of isolates from children. Among the 10 regions, we found 83% (30/36) of the isolates had patterns indistinguishable from isolates from local pigs and dogs (Technical Appendix Figure 1). The rest of the isolates (6/36) shared patterns with those from pigs from other regions (Technical Appendix Figure 2).

## Discussion

*Y. enterocolitica* is a zoonotic pathogen widely distributed throughout China. However, yersiniosis, predominantly a diarrheal illness, is not notifiable through the national surveillance systems of China. Our large-scale investigation of *Y. enterocolitica* infection among children ≤5 years of age with diarrhea in China found *Y. enterocolitica* bioserotype 3/O:3 is a major pathogen (prevalence 0.56%; 41/7,304). According to reports in various years from Finland, Canada, Chile, Holland, Italy, New Zealand, and the United States, the prevalence of *Y. enterocolitica* among patients with diarrhea was ≈0.6%–2.9% (*24*–*28*).

Most hospitals in China do not routinely test for *Y. enterocolitica*; diagnosis of diarrhea is mainly based on signs, symptoms, and fecal microscopy results. We found that a predominant characteristic of feces from young children with *Y. enterocolitica* infection was the presence of leukocytes (Figure 4), which were detectable despite the consistency of the fecal samples (Figure 3, panel A). However, the presence of fecal leukocytes is often regarded as a diagnostic feature of bacillary dysentery, a term that is used interchangeably with shigellosis, and consequently diagnosed as such, leading to confusion over which pathogen is the causative agent (*Shigella*, *Salmonella*, enteroinvasive *Escherichia coli*, *Campylobacter*, or *Yersinia*) (*29*). A decade (2004–2013) of surveillance in Beijing indicates that bacillary dysentery consistently ranked as the infectious disease of the highest incidence, except for a second place ranking in 2013, in which bacillary dysentery was 3–6-fold the national average incidence (*29*). The primary reason for the overdiagnosis of shigellosis has been the lack of microbiological diagnostic testing. In this study, according to the primary diagnoses listed on the questionnaires, quite a few cases among children were regarded as shigellosis. Conversely, diarrhea cases without fecal leukocytes tended not to be diagnosed as infectious diarrhea, which delayed administration of the correct and best treatments. 

A limitation of our study was that fecal microscopy could not be conducted in some village clinics. Whether these children without fecal microscopy results were overlooked requires further investigation. 

In countries where clinical signs guide diagnosis, a case of diarrhea with persistent abdominal pain and fever would prompt culture for *Y. enterocolitica* and cold enrichment (*30*). Using microbiological diagnostic techniques, we found that the prevalence of pathogenic *Y. enterocolitica* among children <5 years of age with diarrhea (0.61%) surpassed that of *Shigella* species in some regions (0.14%; data not shown).

Reports from some countries have shown the prevalence of pathogenic *Y. enterocolitica* infection among children to be higher and the prevalence of nonpathogenic strains to be lower than that among adults (*31*), which is consistent with our study. In Beijing, the prevalence of pathogenic *Y. enterocolitica* among children <5 years of age with diarrhea was ≈6-fold higher than that among adults with diarrhea, and the prevalence of infection with biotype 1A was the reverse (≈6-fold higher among adults than among children <5 years of age with diarrhea). Besides other possible explanations, such as incidental infection or acquired immunity, misuse of antimicrobial drugs by adults might play a substantial role in limiting infection with pathogenic strains among adults in China; isolation of pathogenic strains from adult patients is generally difficult. However, a typical family in China would not readily administer antimicrobial drugs to young children. In this study, primary hospitals given the opportunity to be sentinel sites for *Y. enterocolitica* isolation were instructed to avoid giving patients antimicrobial drugs before enrollment as much as possible.

Biotype 1A is a *Y. enterocolitica* strain widely distributed throughout the natural environment that serves as a source of infection and food contamination (*32*). The diets of adults are not as restricted as that of children, which potentially explains why a higher percentage of adults have diarrhea attributable to biotype 1A. Biotype 1A isolates have generally been regarded as avirulent, but some isolates harboring genes such as *ystB*, which encodes a heat-stable enterotoxin, have been implicated in foodborne and nosocomial outbreaks (*6*–*8*). In this study, *ystB* was present in most biotype 1A isolates found from adults, suggesting possible pathogenicity of these isolates as well.

This study had another limitation. The diagnostics protocol included a cold enrichment step, which made identifying nonpathogenic strains and inapparent infections more likely and diagnosis more time-consuming (*33*). Consequently, early treatment decisions could not be guided by our diagnostic test results. However, cold enrichment did improve overall recovery of *Y. enterocolitica*, especially when the bacteria density of the fecal sample was low, such as during the convalescent phase or long-term shedding. Diarrhea is often considered to be mild and self-limiting in patients with *Y. enterocolitica* infection (*5*), but we found 3 cases of long-term bacterial shedding of *Y. enterocolitica* 3/O:3 among children. Low acquired immunity among children might be a possible explanation, and a timely and accurate diagnosis is greatly needed to prevent these types of cases from occurring. Although cold enrichment has its limitations, we included it in the protocol to more accurately and completely diagnose *Y. enterocolitica* infection in the study population. This method has been used in multiple surveillance studies around the world (*12*,*13*,*34*–*36*).

Generally, only a subset of bioserotypes are pathogenic, mainly 1B/O:8; 4/O:3; 2/O:5,27; 2/O:9; and 3/O:3 (mostly found in China). In recent decades in most countries and regions, the pathogenic bioserotypes of highest prevalence and incidence shifted from strain 1B/O:8 to 4/O:3. In China, the shift was from 2/O:9 to 3/O:3; as of July 2017, the 1B/O:8 strain has not been detected yet in China. Strain 4/O:3, having limited PFGE pattern diversity and high similarity with reference strains abroad (data not shown), has rarely been isolated in China. Only a single 4/O:3 isolate was found in this study, even though this strain is the predominant bioserotype found in other parts of the world. Whether this strain was acquired domestically or from travelers to China is not known. According to our previous research (*37*), the susceptibilities of strains 3/O:3 and 4/O:3 to O:3-specific phage are similar; thus, O:3-specific phage susceptibility cannot explain the rarity of 4/O:3 in China, but susceptibility to 4/O:3-specific phage might.

When comparing pathogenic isolates from different sources, isolates from children shared PFGE patterns with isolates from local pigs and dogs, suggesting a link between isolates from animals and human infection. Pigs have been shown to be a source of *Y. enterocolitica* infection (*20*,*38*–*41*). In correlation studies in Belgium and Norway, human infections have been associated with ingestion of raw or undercooked pork (*38*,*39*). In Germany, the state with the highest consumption of meat showed the highest incidence of yersiniosis (*20*). The prevalence of pathogenic *Y. enterocolitica* was even higher in China than in Europe, potentially because the population of China is a big consumer of pork (*15*). However, persons in China seldom eat undercooked pork; a more likely route of transmission is cross-contamination (*12*). Lee et al. described cases in which *Yersinia* seemed to have been transferred from raw tripe to infants on the unwashed hands of caregivers (*42*). Whether transmission is aided by transportation of pork products between regions needs further investigation. Pigs from multiple regions are slaughtered in Beijing, the location where we found the highest number of isolates from children with PFGE patterns indistinguishable from isolates from pigs. Researchers in Japan reported isolation of *Y. enterocolitica* of different bioserotypes from imported meat products (i.e., pork, beef, and chicken) from Europe, the United States, and other regions of Asia (*43*). PFGE patterns of some isolates from children in our study were not indistinguishable from those from animals, perhaps because our surveillance of isolates from animals is not complete.

The results of this nationwide investigation in China emphasize that *Y. enterocolitica* bioserotype 3/O:3 is a prominent pathogen of children <5 years of age with diarrhea and that microbiological diagnostic testing should be considered for patients who have leukocytes in their feces. Children might acquire infection from contaminated food, and to establish an epidemiologic link between the illness and the consumption of or contact with pork, a case–control study comparing exposures of ill and healthy children is needed. Geographic or seasonal differences in prevalence should also be examined in the future. Our team will continue its surveillance of *Y. enterocolitica* infection among children with diarrhea. We suggest that hospitals routinely test for *Y. enterocolitica* and report laboratory-confirmed cases to public health authorities.

**Technical Appendix.** Comparison of pulse-field gel electrophoresis patterns of *Yersinia enterocolitica* 3/O:3 isolates from children <5 years of age, pigs, and dogs from different regions of China, 2010–2015.

## References

[1] Trček J, Fuchs TM, Trülzsch K. Analysis of *Yersinia enterocolitica* invasin expression *in vitro* and i*n vivo* using a novel *luxCDABE* reporter system. Microbiology. 2010;156:2734–45. 10.1099/mic.0.038240-0 20558509

[2] Bottone EJ. *Yersinia enterocolitica*: the charisma continues. Clin Microbiol Rev. 1997;10:257–76. 9105754 10.1128/cmr.10.2.257 PMC172919

[3] European Food Safety Authority; European Centre for Disease Prevention and Control. The European Union summary report on trends and sources of zoonoses, zoonotic agents and food-borne outbreaks in 2013. EFSA J. 2015;13:3991. 10.2903/j.efsa.2015.3991

[4] Ong KL, Gould LH, Chen DL, Jones TF, Scheftel J, Webb TH, et al. Changing epidemiology of *Yersinia enterocolitica* infections: markedly decreased rates in young black children, Foodborne Diseases Active Surveillance Network (FoodNet), 1996-2009. Clin Infect Dis. 2012;54(Suppl 5):S385–90. 10.1093/cid/cis053 22572658 PMC4593613

[5] Cover TL, Aber RC. Yersinia enterocolitica. N Engl J Med. 1989;321:16–24. 10.1056/NEJM198907063210104 2659991

[6] Singh I, Virdi JS. Production of *Yersinia* stable toxin (YST) and distribution of *yst* genes in biotype 1A strains of *Yersinia enterocolitica.* J Med Microbiol. 2004;53:1065–8. 10.1099/jmm.0.45527-0 15496381

[7] Burnens AP, Frey A, Nicolet J. Association between clinical presentation, biogroups and virulence attributes of *Yersinia enterocolitica* strains in human diarrhoeal disease. Epidemiol Infect. 1996;116:27–34. 10.1017/S0950268800058921 8626001 PMC2271249

[8] Bhagat N, Virdi JS. The enigma of *Yersinia enterocolitica* biovar 1A. Crit Rev Microbiol. 2011;37:25–39. 10.3109/1040841X.2010.506429 20883083

[9] Valentin-Weigand P, Heesemann J, Dersch P. Unique virulence properties of *Yersinia enterocolitica* O:3—an emerging zoonotic pathogen using pigs as preferred reservoir host. Int J Med Microbiol. 2014;304:824–34. 10.1016/j.ijmm.2014.07.008 25172222

[10] Wang X, Qiu H, Jin D, Cui Z, Kan B, Xiao Y, et al. O:8 serotype *Yersinia enterocolitica* strains in China. Int J Food Microbiol. 2008;125:259–66. 10.1016/j.ijfoodmicro.2008.04.016 18541322

[11] Fukushima H, Hao Q, Wu K, Hu X, Chen J, Guo Z, et al. *Yersinia enterocolitica* O9 as a possible barrier against *Y. pestis* in natural plague foci in Ningxia, China. Curr Microbiol. 2001;42:1–7. 10.1007/s002840010168 11116388

[12] Wang X, Cui Z, Jin D, Tang L, Xia S, Wang H, et al. Distribution of pathogenic *Yersinia enterocolitica* in China. Eur J Clin Microbiol Infect Dis. 2009;28:1237–44. 10.1007/s10096-009-0773-x 19575249

[13] Wang X, Cui Z, Wang H, Tang L, Yang J, Gu L, et al. Pathogenic strains of *Yersinia enterocolitica* isolated from domestic dogs (*Canis familiaris*) belonging to farmers are of the same subtype as pathogenic *Y. enterocolitica* strains isolated from humans and may be a source of human infection in Jiangsu Province, China. J Clin Microbiol. 2010;48:1604–10. 10.1128/JCM.01789-09 20181899 PMC2863899

[14] Fukushima H, Shimizu S, Inatsu Y. *Yersinia enterocolitica* and *Yersinia pseudotuberculosis* detection in foods. J Pathogens. 2011;2011:735308. 10.4061/2011/735308 22567341 PMC3335482

[15] Liang J, Wang X, Xiao Y, Cui Z, Xia S, Hao Q, et al. Prevalence of *Yersinia enterocolitica* in pigs slaughtered in Chinese abattoirs. Appl Environ Microbiol. 2012;78:2949–56. 10.1128/AEM.07893-11 22327599 PMC3318836

[16] Funk JA, Troutt HF, Davis SA, Fossler CP. In vitro susceptibility of *Yersinia enterocolitica* isolated from the oral cavity of swine. J Food Prot. 2000;63:395–9. 10.4315/0362-028X-63.3.395 10716571

[17] Jones TF, Buckingham SC, Bopp CA, Ribot E, Schaffner W. From pig to pacifier: chitterling-associated yersiniosis outbreak among black infants. Emerg Infect Dis. 2003;9:1007–9. 10.3201/eid0908.030103 12967503 PMC3020614

[18] Chakraborty A, Komatsu K, Roberts M, Collins J, Beggs J, Turabelidze G, et al. The descriptive epidemiology of yersiniosis: a multistate study, 2005-2011. Public Health Rep. 2015;130:269–77. 10.1177/003335491513000314 25931631 PMC4388225

[19] Scallan E, Hoekstra RM, Angulo FJ, Tauxe RV, Widdowson M-A, Roy SL, et al. Foodborne illness acquired in the United States—major pathogens. Emerg Infect Dis. 2011;17:7–15. 10.3201/eid1701.P11101 21192848 PMC3375761

[20] Rosner BM, Stark K, Werber D. Epidemiology of reported *Yersinia enterocolitica* infections in Germany, 2001-2008. BMC Public Health. 2010;10:337. 10.1186/1471-2458-10-337 20546575 PMC2905328

[21] Kotloff KL, Nataro JP, Blackwelder WC, Nasrin D, Farag TH, Panchalingam S, et al. Burden and aetiology of diarrhoeal disease in infants and young children in developing countries (the Global Enteric Multicenter Study, GEMS): a prospective, case-control study. Lancet. 2013;382:209–22. 10.1016/S0140-6736(13)60844-2 23680352

[22] Huang Y, Wang X, Cui Z, Yang Y, Xiao Y, Tang L, et al. Possible use of *ail* and *foxA* polymorphisms for detecting pathogenic *Yersinia enterocolitica.* BMC Microbiol. 2010;10:211. 10.1186/1471-2180-10-211 20691098 PMC2924855

[23] Wauters G. Contribution à l'étude de *Yersinia enterocolitica*. Belgium: Vander Louvain; 1970.

[24] Morris JG Jr, Prado V, Ferreccio C, Robins-Browne RM, Bordun AM, Cayazzo M, et al. *Yersinia enterocolitica* isolated from two cohorts of young children in Santiago, Chile: incidence of and lack of correlation between illness and proposed virulence factors. J Clin Microbiol. 1991;29:2784–8. 1757549 10.1128/jcm.29.12.2784-2788.1991 PMC270433

[25] Fenwick SG, McCarthy MD. *Yersinia enterocolitica* is a common cause of gastroenteritis in Auckland. N Z Med J. 1995;108:269–71. 7637924

[26] Stolk-Engelaar VM, Hoogkamp-Korstanje JA. Clinical presentation and diagnosis of gastrointestinal infections by *Yersinia enterocolitica* in 261 Dutch patients. Scand J Infect Dis. 1996;28:571–5. 10.3109/00365549609037963 9060059

[27] Zaidi AK, Macone A, Goldmann AD. Impact of simple screening criteria on utilization of low-yield bacterial stool cultures in a Children’s Hospital. Pediatrics. 1999;103:1189–92. 10.1542/peds.103.6.1189 10353927

[28] Fredriksson-Ahomaa M. *Yersinia enterocolitica* and *Yersinia pseudotuberculosis*. In: Simjee S, editor. Foodborne diseases. Totowa (New Jersey): Humana Press; 2007. p. 79–113.

[29] Wang X, Wang J, Sun H, Xia S, Duan R, Liang J, et al. Etiology of childhood infectious diarrhea in a developed region of China: compared to childhood diarrhea in a developing region and adult diarrhea in a developed region. PLoS One. 2015;10:e0142136. 10.1371/journal.pone.0142136 26528820 PMC4631449

[30] Guerrant RL, Van Gilder T, Steiner TS, Thielman NM, Slutsker L, Tauxe RV, et al.; Infectious Diseases Society of America. Practice guidelines for the management of infectious diarrhea. Clin Infect Dis. 2001;32:331–51. 10.1086/318514 11170940

[31] Huovinen E, Sihvonen LM, Virtanen MJ, Haukka K, Siitonen A, Kuusi M. Symptoms and sources of *Yersinia enterocolitica*-infection: a case-control study. BMC Infect Dis. 2010;10:122. 10.1186/1471-2334-10-122 20487529 PMC2883546

[32] Stephan R, Joutsen S, Hofer E, Säde E, Björkroth J, Ziegler D, et al. Characteristics of *Yersinia enterocolitica* biotype 1A strains isolated from patients and asymptomatic carriers. Eur J Clin Microbiol Infect Dis. 2013;32:869–75. 10.1007/s10096-013-1820-1 23354676

[33] Schmitz AMTR. *Yersinia enterocolitica* infections. Epidemiology and control. In: Brachman PS, Abrutyn E, editors. Bacterial infections of humans. New York: Springer; 2009. p. 939–57.

[34] Mingrone MG, Fantasia M, Figura N, Guglielmetti P. Characteristics of *Yersinia enterocolitica* isolated from children with diarrhea in Italy. J Clin Microbiol. 1987;25:1301–4. 3611321 10.1128/jcm.25.7.1301-1304.1987 PMC269198

[35] Galanakis E, Perdikogianni C, Maraki S, Giannoussi E, Kalmanti M, Tselentis Y. Childhood *Yersinia enterocolitica* infection in Crete. Eur J Clin Microbiol Infect Dis. 2006;25:65–6. 10.1007/s10096-005-0058-y 16391914

[36] El Qouqa IA, El Jarou MA, Samaha AS, Al Afifi AS, Al Jarousha AM. *Yersinia enterocolitica* infection among children aged less than 12 years: a case-control study. Int J Infect Dis. 2011;15:e48–53. 10.1016/j.ijid.2010.09.010 21131221

[37] Liang J, Li X, Zha T, Chen Y, Hao H, Liu C, et al. DTDP-rhamnosyl transferase RfbF, is a newfound receptor-related regulatory protein for phage phiYe-F10 specific for *Yersinia enterocolitica* serotype O:3. Sci Rep. 2016;6:22905. 10.1038/srep22905 26965493 PMC4786787

[38] Tauxe RV, Vandepitte J, Wauters G, Martin SM, Goossens V, De Mol P, et al. *Yersinia enterocolitica* infections and pork: the missing link. Lancet. 1987;1:1129–32. 10.1016/S0140-6736(87)91683-7 2883453

[39] Ostroff SM, Kapperud G, Hutwagner LC, Nesbakken T, Bean NH, Lassen J, et al. Sources of sporadic *Yersinia enterocolitica* infections in Norway: a prospective case-control study. Epidemiol Infect. 1994;112:133–41. 10.1017/S0950268800057496 8119353 PMC2271483

[40] Korte T, Fredriksson-Ahomaa M, Niskanen T, Korkeala H. Low prevalence of *yadA*-positive *Yersinia enterocolitica* in sows. Foodborne Pathog Dis. 2004;1:45–52. 10.1089/153531404772914455 15992261

[41] Fredriksson-Ahomaa M, Stolle A, Stephan R. Prevalence of pathogenic *Yersinia enterocolitica* in pigs slaughtered at a Swiss abattoir. Int J Food Microbiol. 2007;119:207–12. 10.1016/j.ijfoodmicro.2007.07.050 17727997

[42] Lee LA, Gerber AR, Lonsway DR, Smith JD, Carter GP, Puhr ND, et al. *Yersinia enterocolitica* O:3 infections in infants and children, associated with the household preparation of chitterlings. N Engl J Med. 1990;322:984–7. 10.1056/NEJM199004053221407 2314448

[43] Fukushima H, Hoshina K, Itogawa H, Gomyoda M. Introduction into Japan of pathogenic *Yersinia* through imported pork, beef and fowl. Int J Food Microbiol. 1997;35:205–12. 10.1016/S0168-1605(96)01223-8 9105929

