# Supplementary material for: Prevalence of Yersinia enterocolitica Bioserotype 3/O:3 among Children with Diarrhea, China, 2010–2015
Source: Emerg Infect Dis. 2017 Sep;23(9):1502–9. doi: 10.3201/eid2309.160827 (PMC5572862; doi:10.3201/eid2309.160827)
Supplement: Supplementary file 1 — Technical Appendix. Comparison of pulse-field gel electrophoresis patterns of Yersinia enterocolitica 3/O:3 isolates from children <5 years of age, pigs, and dogs from different regions of China, 2010–2015. [file 16-0827-Techapp-s1.pdf]

Article DOI: https://doi.org/10.3201/eid2309.160827

Prevalence of *Yersinia enterocolitica* Bioserotype 3/O:3 among Children with Diarrhea, China, 2010–2015

Technical Appendix

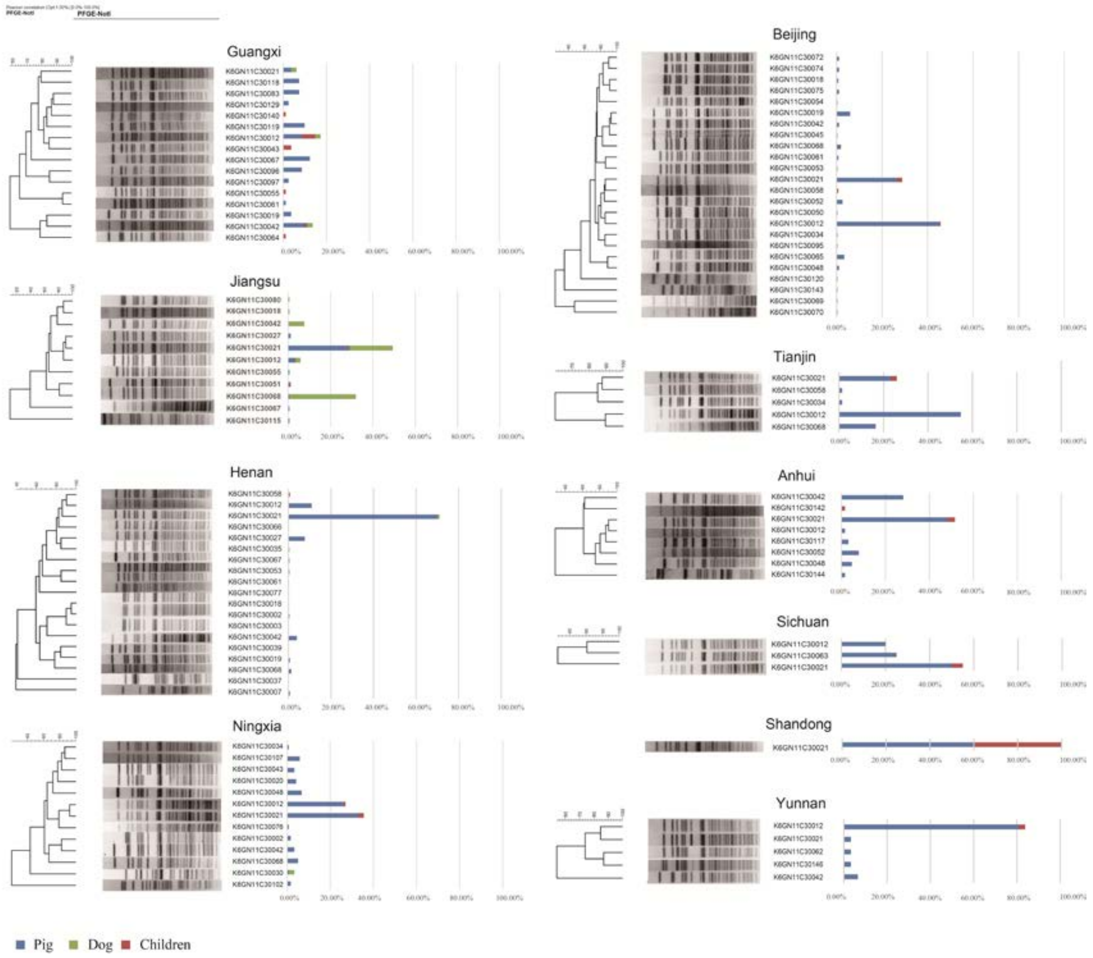

Technical Appendix Figure 1. Cluster analysis of pulsed-field gel electrophoresis patterns of 3/O:3 *Yersinia enterocolitica* isolates obtained from children ≤5 years of age, pigs, and dogs by region, China,

2010–2015. Gels depict DNA digests with *Not*I. Clustering trees indicate relatedness of strains. The proportion of each pattern detected per region for children, dogs, and pigs are indicated.

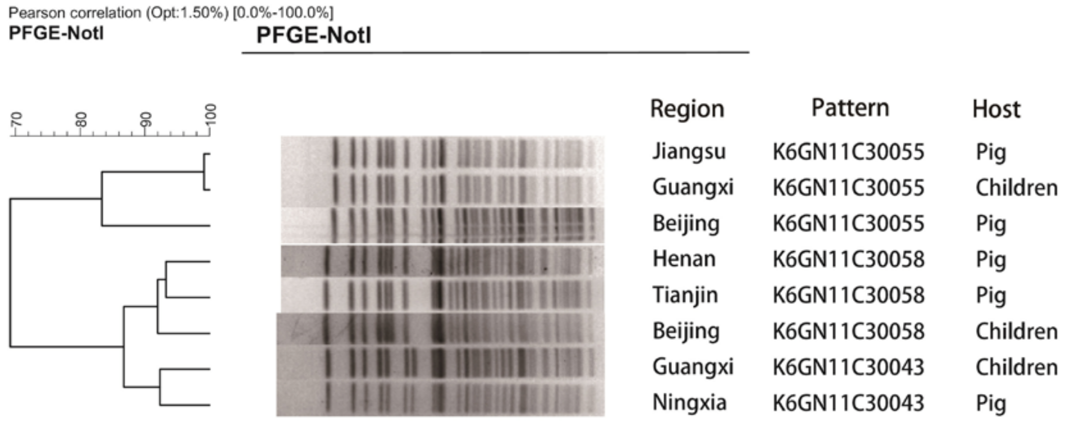

Technical Appendix Figure 2. Cluster analysis of pulsed-field gel electrophoresis patterns show that 3/O:3 *Yersinia enterocolitica* isolates from 6 children <u><</u>5 years of age from Guangxi and Beijing share patterns indistinguishable from isolates found in pigs from other regions. Gels depict DNA digests with *Not*I, and clustering trees indicate the relatedness of the isolates shown.
